# GPT-4 performance on querying scientific publications: reproducibility, accuracy, and impact of an instruction sheet

**DOI:** 10.1186/s12874-024-02253-y

**Published:** 2024-06-25

**Authors:** Kaiming Tao, Zachary A. Osman, Philip L. Tzou, Soo-Yon Rhee, Vineet Ahluwalia, Robert W. Shafer

**Affiliations:** 1 Division of Infectious Diseases, Department of Medicine, Stanford University, Stanford, CA USA; 2 Aphorism Labs, Palo Alto, CA USA

**Keywords:** Large language model, HIV drug resistance, Systematic review, GPT-4, Data extraction

## Abstract

**Background:**

Large language models (LLMs) that can efficiently screen and identify studies meeting specific criteria would streamline literature reviews. Additionally, those capable of extracting data from publications would enhance knowledge discovery by reducing the burden on human reviewers.

**Methods:**

We created an automated pipeline utilizing OpenAI GPT-4 32 K API version “2023–05-15” to evaluate the accuracy of the LLM GPT-4 responses to queries about published papers on HIV drug resistance (HIVDR) with and without an instruction sheet. The instruction sheet contained specialized knowledge designed to assist a person trying to answer questions about an HIVDR paper. We designed 60 questions pertaining to HIVDR and created markdown versions of 60 published HIVDR papers in PubMed. We presented the 60 papers to GPT-4 in four configurations: (1) all 60 questions simultaneously; (2) all 60 questions simultaneously with the instruction sheet; (3) each of the 60 questions individually; and (4) each of the 60 questions individually with the instruction sheet.

**Results:**

GPT-4 achieved a mean accuracy of 86.9% – 24.0% higher than when the answers to papers were permuted. The overall recall and precision were 72.5% and 87.4%, respectively. The standard deviation of three replicates for the 60 questions ranged from 0 to 5.3% with a median of 1.2%. The instruction sheet did not significantly increase GPT-4’s accuracy, recall, or precision. GPT-4 was more likely to provide false positive answers when the 60 questions were submitted individually compared to when they were submitted together.

**Conclusions:**

GPT-4 reproducibly answered 3600 questions about 60 papers on HIVDR with moderately high accuracy, recall, and precision. The instruction sheet's failure to improve these metrics suggests that more sophisticated approaches are necessary. Either enhanced prompt engineering or finetuning an open-source model could further improve an LLM's ability to answer questions about highly specialized HIVDR papers.

**Supplementary Information:**

The online version contains supplementary material available at 10.1186/s12874-024-02253-y.

## Background

The systematic review of data from multiple research studies is often required to answer many of the most significant biomedical questions. However, the literature searches required for a systematic review often suffer from low sensitivity (recall) and specificity (precision) in part as a result of the limitations of current search tools which rely on the Medical Subject Headings (MeSH) key words, the National Library of Medicine’s controlled vocabulary used for indexing articles [1]. Extracting data from relevant studies also requires painstaking review by highly trained human reviewers.

The use of automated software tools to assist in reviewing research papers has become a topic of increasing interest. Most such tools have used natural language processing (NLP) and machine learning (ML) algorithms primarily to screen the titles and abstracts of publications to determine whether they meet the search criteria for a systematic review [2–8]. Several studies have also described the potential for using the representational language model Bidirectional Encoder Representations from Transformers (BERT) and the Generative Pre-trained Transformer (GPT) large language models (LLMs) for reviewing the full text of published studies [9–12]. LLMs have also been evaluated for their ability to summarize research studies [13, 14].

We have extensive experience reviewing published studies on the topic of human immunodeficiency virus (HIV) drug resistance having maintained the Stanford HIV Drug Resistance Database (HIVDB; https://hivdb.stanford.edu) and performed multiple systematic literature reviews [15, 16]. In this study, we evaluated the ability of GPT-4 to correctly answer questions about publications on HIV drug resistance with and without an instruction sheet designed to provide GPT-4 with specialized HIV drug resistance knowledge. We evaluated publications considered for inclusion in a curated database. This database primarily links mutations in the genetic targets of HIV therapy to the antiviral treatments of the persons from whom the sequences were obtained and to the impact of these mutations on the i*n vitro* susceptibility to individual HIV drugs.

## Methods

### HIV drug resistance questions

We designed 60 questions pertaining to HIV drug resistance reflecting the type of information typically extracted from published papers evaluated for possible addition to HIVDB. Most of the questions dealt with linking HIV genetic sequence data to two other forms of data: (1) the antiviral treatments received by the patients from whom the sequenced viruses were obtained and (2) the effect of mutations in these viruses on their susceptibility to antiviral drugs. The questions were of three types: Boolean, requiring yes or no answers; numerical, where the correct response was an integer; and list-based, where a series of items constituted the correct answer. The complete list of questions can be found in Supplementary File 1.

### Published papers

We selected 60 published papers on HIV drug resistance identified in recent PubMed searches and in recent GenBank database submissions including 19 published after September 2021, the cut-off date for the dataset used to train the GPT-4 model that was used. Nearly two-thirds of the papers reported HIV genotypic resistance data (e.g., genetic sequence data or lists of HIV drug-resistance mutations). Nearly one-half reported that their sequences had been submitted to GenBank, the standard public repository for sequence data, and provided GenBank accession numbers. The selected papers often reported the antiviral treatment histories of patients undergoing virus sequencing, the samples submitted for sequencing, the technology used for sequencing, and the results of in vitro susceptibility testing. Two authors reviewed each paper to determine the answers to the 60 questions. A third author designated the correct answer when there was a disagreement between the first two authors. The complete list of papers can be found in Supplementary File 2.

### Instruction sheet

The instruction sheet contained 2002 words that provided background knowledge about HIV drug resistance and the type of information that a human curator would need to know to identify the relevant data for inclusion in HIVDB (Supplementary File 3). This document encapsulated fundamental antiviral therapy and HIV drug resistance concepts, alongside a description of frequently used terms and abbreviations within the field. The instruction sheet was not designed to be a comprehensive treatise on antiviral therapy and HIV drug resistance but rather to offer practical guidance to human curators with some background HIV knowledge. The instruction sheet contained information considered useful to answering many of the 60 questions. However, it was not designed specifically to answer each of the questions developed for this study.

### Automated query pipeline

**Fig. 1:**
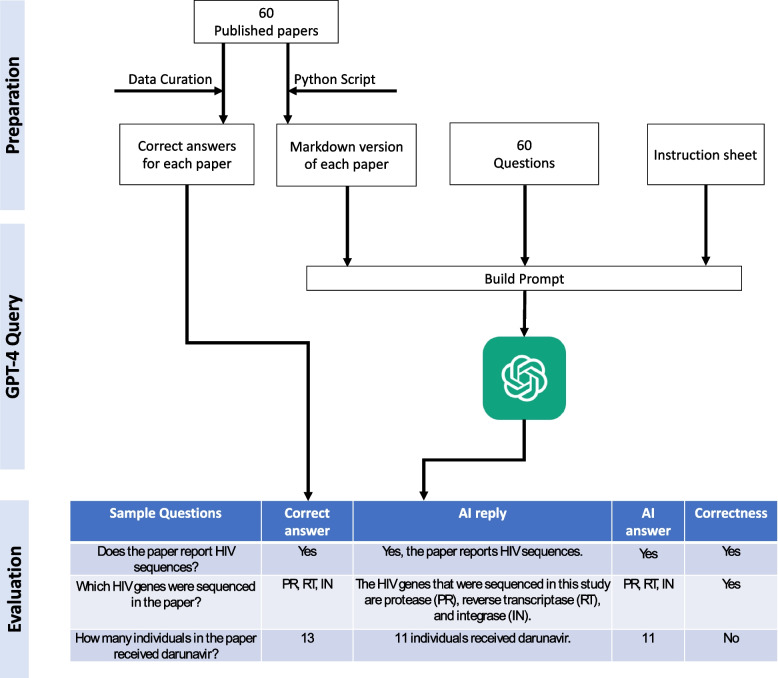
Automated query pipeline work flow. The first step involved developing 60 questions relevant to HIV drug resistance, identifying 60 published papers, and developing an approximately 2000 word instruction sheet with HIV drug resistance information. Each paper was reviewed by two human reviewers and a markdown version of each paper’s full text was created. The second step involved querying GPT-4: building a prompt that included (1) the marked down version of each paper, (2) all 60 questions, and (3) the instruction sheet. The third step evaluated the GPT-4 answers to assess whether they were the same as the answers determined by the human curators. Three sample questions are shown including one for which the correct answer was Yes or No, another for which the correct answer was a list of items, and a third for which the correct answer was a number

We designed an automated pipeline utilizing OpenAI GPT-4 32K API version “2023–05-15” (Microsoft Azure, accessed Sep 15th, 2023) (Fig.1). A Python script was used to transform a published paper to a markdown format containing the text of the study methods, study results, tabular data, and figure legends. The abstract, introduction, discussion, and references were excluded from the markdown version of the paper. The median number of tokens in each markdown paper was 5338 (range:1282 to 13,861). On average, one token is about 0.75 words or about four English language characters. We chose to not submit the introduction, discussion, and references to GPT-4 because these parts of a paper often refer to the work of other studies.

Each GPT-4 query consisted of one markdown paper plus one of the following: (1) all 60 questions presented simultaneously; (2) all 60 questions presented simultaneously with the instruction sheet; (3) each of the 60 questions presented individually; and (4) each of the 60 questions presented individually with the instruction sheet.

We refer to the process of submitting the 60 questions simultaneously as the multiple-question mode and the process of submitting each question individually as the single-question mode. We refer to the process of presenting all questions without the instruction sheet as the base model. The single-question mode necessitated repeatedly submitting the same markdown paper with each question. It was therefore much more time consuming and expensive than the multiple-question mode.

If GPT-4 failed to answer all 60 questions for a paper or if a time-out error occurred when questions were presented in the multiple-question mode, the unanswered questions were resubmitted along with the paper.

Supplementary File 4 provides an example GPT-4 prompt. Supplementary File 5 provides the Python code used to generate the GPT-4 prompts.

### Automated response evaluation pipeline

We evaluated the accuracy of GPT-4 responses using the following approach: (1) for Boolean questions, a script was used to determine if the response began with “yes” or “no”; (2) for numerical questions, a script was used to determine if the response contained a single number; (3) all other responses were evaluated manually. Accuracy was defined as concordance between the correct answer and the GPT-4 response for Boolean and numerical questions. For list questions, we considered the GPT-4 response to be accurate if it identified at least one element of the correct list. The response was considered inaccurate if it did not identify any element of the correct list or if it identified elements that were not part of the correct list. A manual review of half of the responses to the Boolean and numerical questions confirmed that the script used to determine whether the response began with “yes” or “no” or contained a single number accurately gauged GPT-4’s answers to these questions.

### Experimental design and analyses

To evaluate the performance of GPT-4 in answering questions about a paper, we designed a series of experiments: (1) We assessed the reproducibility of the base model in the multiple-question mode by performing each query in triplicate. (2) We calculated the recall, precision, and F1 score – the harmonic mean of precision and recall, calculated as 2 x (recall * precision) / (recall + precision) – for the base model in multiple-question mode. This analysis was performed on the median of the triplicate results and it was performed separately for results obtained with and without the instruction sheet. (3) We compared the accuracy – measured as the proportion of correct answers – of the base model in the multiple-question mode to its performance when the responses were from randomly permuted papers. In essence, we assessed the accuracy of GPT-4's responses to the submitted paper compared with its accuracy when the answers were drawn from ten randomly selected papers, distinct from the actual paper. (4) We compared the accuracy of the base model in the multiple-question mode to the accuracy with the instruction sheet in the multiple-question mode. (5) Finally, we compared the accuracy of the base model in the multiple-question mode with the accuracy of the base model in the single-question mode, also in triplicate.

## Results

**Fig. 2:**
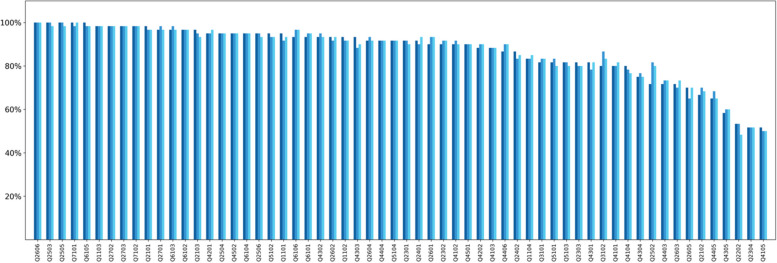
Triplicate determinations of the accuracy of each of the 60 questions applied to each of the 60 papers in the multiple question mode (i.e., all 60 questions presented simultaneously) without the instruction sheet (i.e., base model). The Y-axis indicates the percentage of times in which the GPT4 response was accurate across the 60 papers. The X-axis shows the question ID in descending order of median accuracy. The three bars shown for each question ID indicate separate replicates. The median accuracy for the 60 questions over the three replicates was 91.8% (range: 50.7%-100%). The mean accuracy across all questions and all papers were 86.8%, 86.9%, and 87.1%. Different colors mean different replicates

Figure 2 displays triplicate determinations of the accuracy of GPT-4 on each of the 60 questions applied to each of the 60 papers in the multiple-question mode without the instruction sheet (i.e., base model). The median accuracy for the 60 questions over the three replicates was 91.8% (range: 50.7%-100%). The mean accuracy for the 60 questions over the three replicates was 86.9%. The mean accuracies were similar for Boolean (86.6%), numerical (84.7%), and list (90.2%) questions. The standard deviation (SD) of three replicates for the 60 questions ranged from 0 to 5.3% with a median SD of 1.2% across all questions. The coefficient of variation (CV) of three replicates for the 60 questions ranged from 0 to 0.068 with a median CV of 0.012. The maximum difference between any two of the three replicates was 6 for one question, 4 for two questions, and 3 for three questions.

**Fig. 3:**
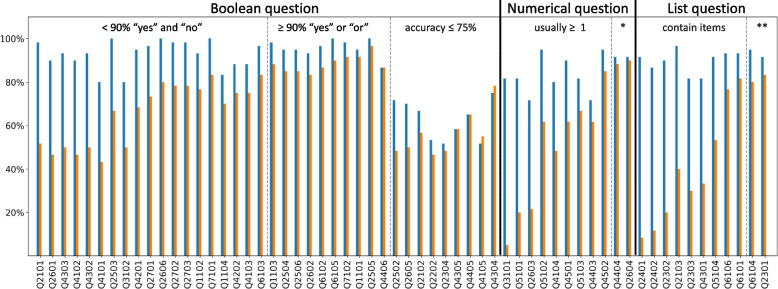
Comparison of the multiple-question base model on the actual paper (blue histograms  ) with the permuted results obtained from different papers (orange histograms  ). The Y-axis indicates the percentage of times in which the answers were correct across the 60 papers. The X-axis shows the question ID. The questions are separated according to whether they are Boolean, numerical, or list questions. The Boolean questions are divided into three sections: (i) questions for which there was a balance between “yes” and “no” correct answers across the 60 papers (i.e., < 90% “yes” or “no”) resulting in a large difference in accuracy after the papers’ answers were permuted; (ii) questions for which ≥ 90% of the answers were “yes” or “no” resulting in little difference in accuracy after the answers were permuted; and (iii) questions for which GPT-4 had an accuracy of about 50% even on the actual paper. The numerical question section separates the two questions for which the correct answer was usually 0 and the list question section separates the two questions for which the answer was usually an empty list. “*” indicates the two numerical questions for which the answer was usually 0. “**” indicates the two list questions for which the answer was usually an empty list

Figure 3 compares the results of one of the three replicates for the base model in multiple-question mode with the results obtained when the answers to the 60 papers were permuted. The mean accuracy for 10 permutations of the papers was 62.9%. Therefore, the increased accuracy of GPT-4 on the actual papers was 24.0% higher than expected by chance on the permuted set of papers (95% CI: 18.6%-29.4%; *p* < 0.000001; paired Student’s t-test). The surprisingly high level of accuracy for permuted answers is explained by the uniformity of responses across many papers. Specifically, for Boolean questions, the answers were not infrequently always ‘yes’ or ‘no.’ Similarly, for numerical questions, the answer was often 0, and for list questions, the answer was often an empty list. Figure 3 demonstrates this in 10 questions where ≥ 90% of the Boolean answers were either ‘yes’ or ‘no’, in two numerical questions where the answer was usually 0, and in two list questions where the answer was usually an empty list.

**Fig. 4:**
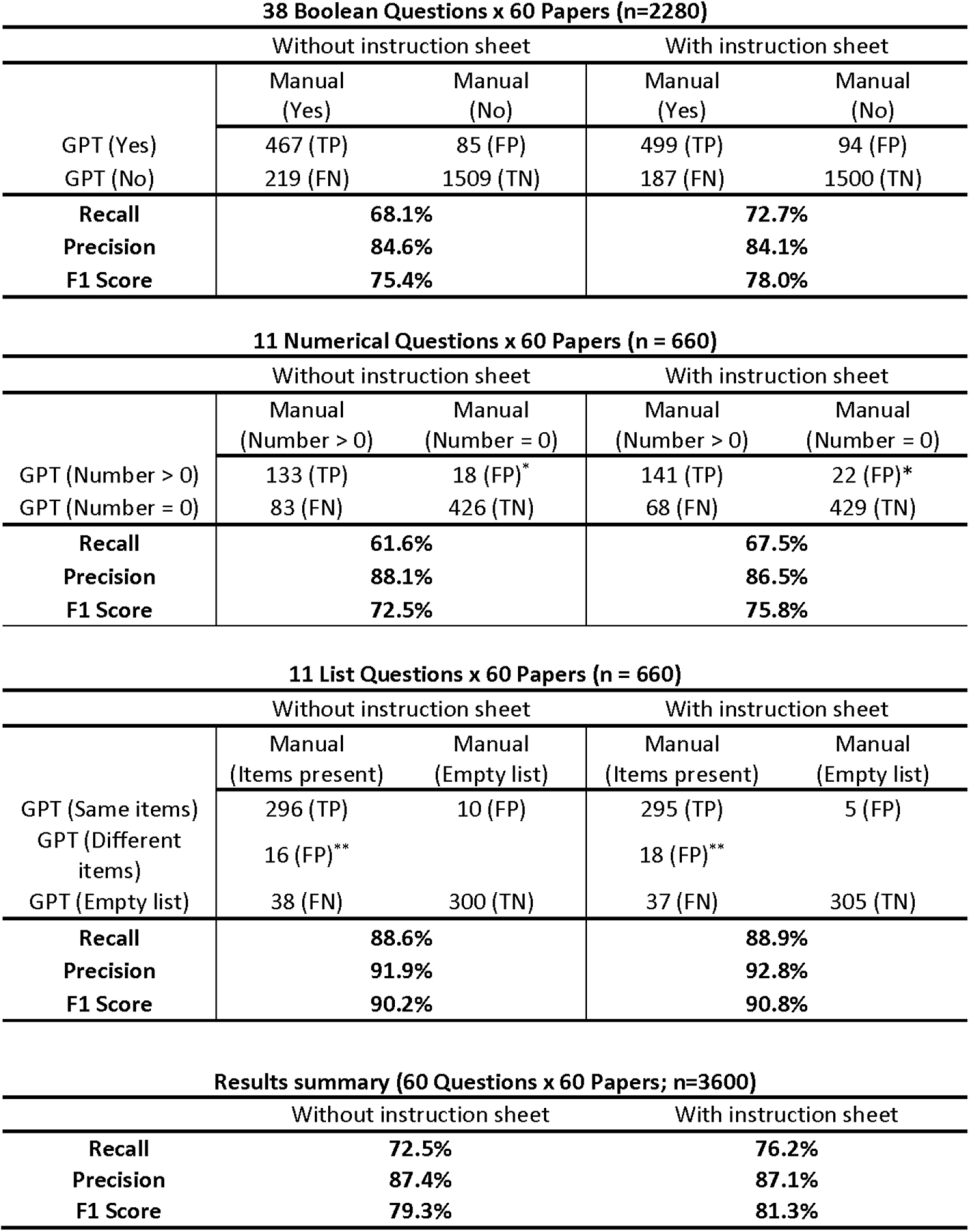
Recall and precision of GPT-4 at answering questions about HIV drug resistance papers: comparison with manual curators. The manual result (obtained by two human curators and a third to break ties) was considered to be the correct answer. Each entry in the six sections containing raw data represents the median of 3 repeats. *Includes questions for which GPT reported a number > 0 when the correct answer was 0 and questions for which GPT reported an incorrect number (i.e., one that differed from the manual review). **The results were considered to be false positives when GPT-4 identified items not identified by manual review. Additionally, 12 of 16 answers obtained without the instruction sheet and 14 of 18 answers obtained with the instruction sheet were considered to be false negatives because GPT-4 also failed to identify any of the items that were identified by manual review. Abbreviations: GPT (GPT-4), TP (true positive), TN (true negative), FP (false positive), FN (false negative), F1 score = 2 * (Recall * Precision) / (Recall + Precision)

Figure 4 shows the precision, recall, and F1 score with and without the instruction sheet separately for the Boolean, numerical, and list questions. Without the instruction sheet, GPT-4 demonstrated a recall of 68.1% and a precision of 84.6% on the 2280 Boolean questions (i.e., 48 questions × 60 papers); a recall of 61.6% and a precision of 88.1% on the 660 numerical questions (i.e., 11 questions × 60 papers); and a recall of 88.6% and a precision of 91.9% on the 660 list questions (i.e., 11 questions × 60 papers). Of the 296 true positive answers for list questions, 273 (92.2%) were identical to the manual answers whereas 23 (7.8%) contained a subset of the manual answers.

**Fig. 5:**
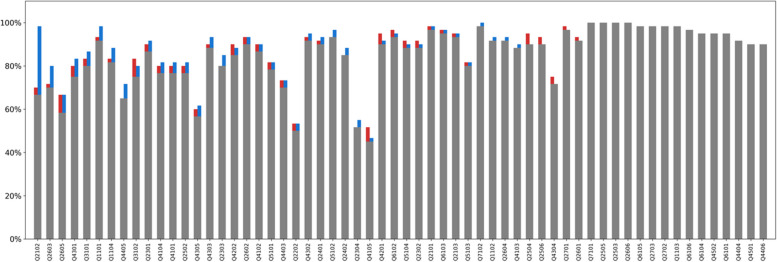
Triplicate determinations of the accuracy of GPT-4 in multiple-question mode with and without the instruction sheet. For each question, two histograms are shown. The left histogram shows the median of triplicate accuracy determinations without the instruction sheet. The right histogram shows the median of triplicate accuracy determinations with the instruction sheet. Increased accuracy associated with the instruction sheet is shown by coloring part of the right-sided histogram in blue while reductions in accuracy are shown by coloring part of the left-sided histogram in red. The sizes of the colored regions indicate the sizes of the increases or decreases in accuracy associated with the instruction sheet. The questions are shown in descending order of the increased accuracy associated with instruction sheet (i.e., the size of the blue histograms)

Figure 5 displays the triplicate determinations of the accuracy of GPT-4 in the multiple-question mode with and without the instruction sheet. Across the 60 questions, the mean net change in accuracy was + 1.2% resulting in an overall accuracy of 88.1% across all questions and papers with the instruction sheet. On average, across the three replicates, the instruction sheet improved the accuracy of 3.2% (*n* = 114) of questions that were initially answered incorrectly. Conversely, 2.0% (*n* = 72) of questions initially answered correctly were incorrect with the instruction sheet. For all 60 questions combined, recall (76.2% vs. 72.5%; *p* = 0.08; Fisher Exact Test) and precision 87.4% vs. 87.1%; NS) were not significantly higher with the instruction sheet compared to without the instruction sheet (Fig. 4).

The instruction sheet significantly impacted three questions: one showed a net accuracy increase of 26.1%, resulting in 16 additional correct responses; another recorded an 8.3% improvement (5 papers); and the third saw a 6.7% increase (4 papers). The remaining questions displayed net changes in accuracy that were no greater or lower than three. The question “Does the paper report GenBank accession numbers for sequenced HIV isolates other than those for laboratory HIV isolates?" was the one associated with a net increased accuracy of 26.1%. The question “How many samples in the paper were reported to have undergone plasma virus sequencing?” was the one associated with a net increased accuracy of 8.3%.

**Fig. 6:**
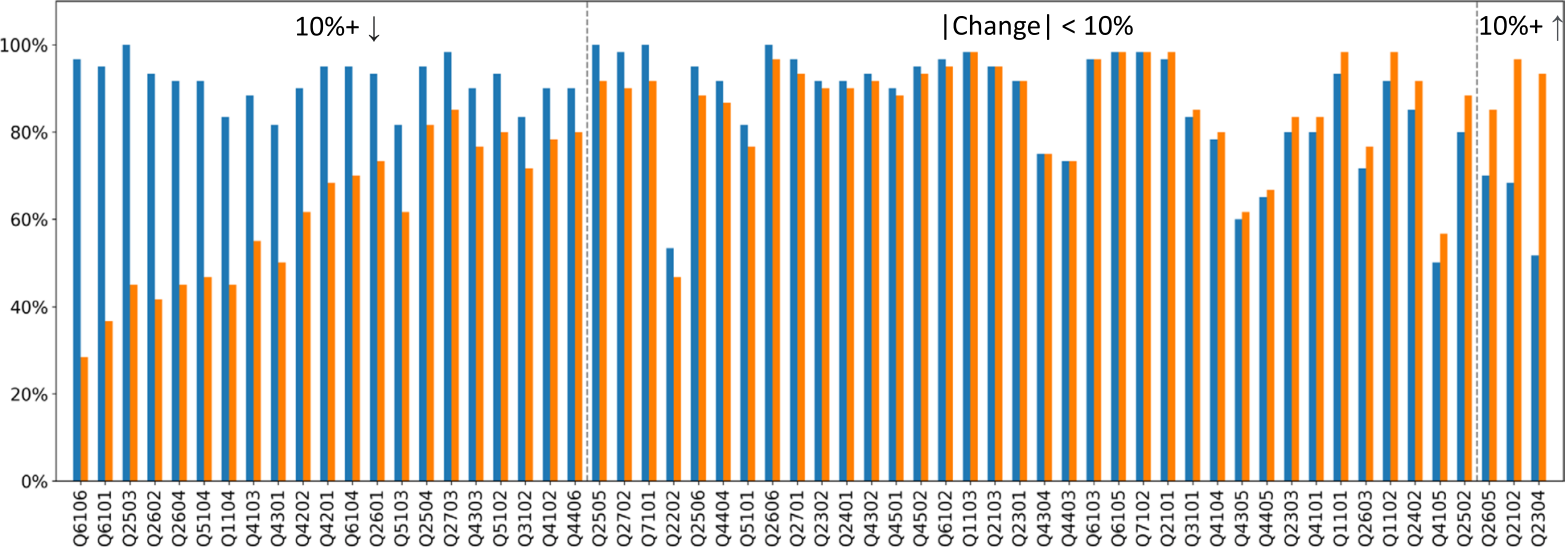
Comparison of the accuracy of the base model in the multiple-question mode (blue histograms  ) and single-question mode (orange histograms  ). The histograms indicate the median of three replicates. The questions are grouped according to the relative accuracy attained with questions presented in the single versus multiple-question mode. Within each panel, the questions are ordered by the difference in accuracy between the multiple and single-question modes. The first panel contains the questions for which the accuracy in the single-question mode was ≥ 10% lower than in the multiple-question mode. The third panel contains the questions for which the accuracy in the single-question mode was ≥ 10% higher than in the multiple-question mode

In an attempt to edit the instruction sheet to increase GPT-4 accuracy for questions that were often answered incorrectly, we modified the query pipeline as follows. Rather than submitting each paper in multiple-question mode, we submitted each paper with just the one question that we were targeting for improvement (i.e., in single-question mode). After running several questions in both modes, we noticed marked differences in GPT-4 accuracy between the multiple-question and single-question modes. Figure 6 compares the accuracy of the multiple-question and single-question mode for all 60 questions without the instruction sheet. Each histogram represents the median of three replicates. Overall, the median and mean accuracy for the single-question mode were significantly lower than the multiple-question mode across all 60 questions: median (83.0% vs 91.8%, *p* = 0.0006; Wilcoxon signed-rank test) and mean (77.6% vs 86.9%, *p* = 0.0005; Wilcoxon signed-rank test).

Figure 6 groups the questions according to whether the accuracy was ≥ 10% lower in the single-question mode (*n* = 21 questions), ≥ 10% higher in the single-question mode (*n* = 3 questions), or less than 10% different between the multiple-question and single-question modes. The largest differences in accuracy between the two modes was for questions for which the answer was usually no for Boolean questions, 0 for numerical questions, and an empty list for list questions. We refer to these usually negative questions as No_0_Empty. Indeed, for the 21 questions that belonged to this category, the median accuracy was 91.0% in the multiple-question mode but only 60.8% in the single-question mode. There was also a strong correlation between the frequency of answers that were No_0_Empty and the reduced accuracy when questions were presented in the single-question mode (*r* = 0.45; *p* = 0.0003).

The cost of using the GPT-4 API to obtain responses for 60 papers × 60 questions in the multiple-question mode without and with the instruction sheet was $240 and $300, respectively. The cost of obtaining responses for 60 papers × 60 questions in the single-question mode without and with the instruction sheet was $1500 and $2000, respectively. The overall cost of this study, considering that most experiments were performed in triplicate was $8120: $240 × 3 plus $300 × 3 plus $1500 × 3 plus $2000 × 1. After completing this study, OpenAI released a new model, GPT-4-turbo (gpt-4–1106-preview), on November 6, 2023. This model significantly reduced costs, decreasing from $0.06 to $0.01 per prompt and from $0.12 to $0.03 per completion. Consequently, the overall cost of this study would have been approximately five times lower.

## Discussion

We submitted the text of the methods, results, tables, and figure legends of 60 published papers on HIV drug resistance together with 60 questions related to HIV drug resistance with and without an instruction sheet to the GPT-4 API. We found that the accuracy of GPT-4 responses was approximately 87%, which was 24% greater than that obtained when the answers to the papers were permuted. With the exception of one question, the accuracy of GPT-4 was not improved with an approximately 2000 word instruction sheet. Notably, GPT-4 was also less likely to answer certain types of questions accurately when they were submitted individually (single-question mode) compared to when they were submitted together (multiple-question mode).

This study differs from most previous studies of automated software tools designed to assist with systematic reviews. First, we prompted the LLM GPT-4 to answer specific questions about entire papers whereas previous studies were often optimized for screening paper title and abstracts [5, 6, 7, 8]. Second, we used GPT without providing training examples, whereas previous studies, were often interactive in that they combined NLP and ML algorithms with user feedback [5, 6, 9, 10, 11]. Finally, the results presented in this study were quantitative and transparent, whereas several previous studies, particularly those using LLMs, presented their results in a qualitative manner.

GPT-4 performs well at summarizing research papers because LLMs are adept at distilling and condensing information into simpler shorter formats [13, 14]. However, answering specific questions can be challenging for LLMs because they only process a limited amount of text at once. This limitation hampers their ability to cross-reference details within longer documents effectively. Indeed, the questions that GPT-4 was likely to answer correctly were those for which the answer could be found in a single paragraph or sentence in a paper. In contrast, those questions that required reasoning about information found in different parts of a paper were less likely to be answered correctly. For example, the question “Does the paper provide complete ART history for all of the individuals in the study?” was answered correctly only about 50% of the time.

The instruction sheet contained information that would have been expected to be helpful for several questions such as “Does the paper report the results of HIV pol sequences?” and “Were the individuals in the study INSTI-naïve?”. Despite this, when GPT-4 was equipped with the information that 'pol' refers to the gene encoding the viral enzymes protease, reverse transcriptase, and integrase, 'INSTI' denotes integrase strand transfer inhibitors, and 'naïve' implies untreated, it still only correctly answered these questions 57% and 62% of the time, respectively.

After completing the experiments outlined in this study, we performed ten queries in an attempt to determine the extent of GPT-4’s HIV drug resistance knowledge. Supplementary File 6 lists each of the ten queries and the entire GPT-4 response (version last updated April 2023). The responses to these additional queries, demonstrated that GPT-4 possesses extensive information about HIV drug resistance. Although the experiments outlined in the results were performed using an earlier version of GPT-4 (last updated September 2021), all of the information included in the GPT-4 response had been publicly available prior to this earlier date. Given that GPT-4 already contained most of the information provided in the instruction sheet, enhancing its performance would likely hinge on providing prompts that demonstrate how to apply its knowledge to a published paper.

In an attempt to edit the instruction sheet to increase GPT-4 accuracy we modified the query pipeline by submitting each paper with just the one question that we were targeting for improvement. This modification led to the study’s second major new finding: when questions were presented individually, GPT-4 tended to provide incorrect affirmative answers to questions that generally warranted a negative response such as ‘no’, ‘0’, or an empty list. For instance, when the query "Which drugs were tested on phenotypic susceptibility in the paper?" was posed separately, there were 40 instances where GPT-4 erroneously referenced drugs that were administered to patients instead of those used in a susceptibility assay. This mistake was infrequent when all 60 questions were asked at once, indicating that presenting the full batch of questions improves GPT-4's understanding of each question's context. The enhanced accuracy observed when presenting multiple questions simultaneously may resemble automatic chain-of-thought prompting [17]. This technique, used in AI interactions, involves supplying step-by-step questions that guide the system through a logical thought sequence, thereby improving its comprehension of complex inquiries.

While enhancing the questions and instruction sheet was of interest, undertaking such revisions methodically would have required an open-ended approach beyond the scope of this study. Nonetheless, we observed that rephrasing two of the questions led to a significantly increased GPT-4 accuracy. For example, the question “Were sequences obtained from individuals with active HIV replication?” was true for 26 of the 60 papers. The median accuracy of GPT-4 on three replicates, with and without the instruction sheet, was 61%. However, the median accuracy of GPT-4 was 97% when we rephrased the question as follows: “Were sequences in the paper obtained from individuals with virological failure while receiving antiretroviral therapy?”. In contrast, the few changes we made to the instruction sheet did not yield substantial increases in GPT-4 accuracy for any of the questions.

## Conclusions

GPT-4 possesses extensive knowledge about HIV drug resistance and it reproducibly answers Boolean, numerical, and list questions about HIV drug resistance papers. Its accuracy, recall, and precision of approximately 87%, 73%, and 87% without human feedback demonstrate its potential at performing this task. GPT-4 faced several challenges beginning with the specialized nature of the questions that were on topics that likely represented a small part of its training corpus [18]. In addition, addressing queries that necessitate making inferences, particularly when dealing with unsaid elements within the text, can be difficult. A more robust familiarity with the subject of HIV drug resistance would potentially have empowered GPT-4 to make better inferences. Finally, the instruction sheet was designed for human comprehension without the multiple examples usually necessary for optimizing a language model’s performance. The inability of GPT-4 to utilize the instruction sheet suggests that more sophisticated prompt engineering approaches or the finetuning of an open source model are likely required to improve accuracy when answering questions on highly specialized research papers.

### Supplementary Information

Supplementary Material 1.

Supplementary Material 2.

Supplementary Material 3.

Supplementary Material 4.

Supplementary Material 5.

Supplementary Material 6.

## Data Availability

All data generated or analyzed during this study are included in this published article and its supplementary information files. 2R24AI13661806. The funder played no role in this review.

## References

[1] Jin Q, Kim W, Chen Q, Comeau DC, Yeganova L, Wilbur WJ (2023). MedCPT: contrastive pre-trained transformers with large-scale PubMed search logs for zero-shot biomedical information retrieval. Bioinformatics..

[2] Cierco Jimenez R, Lee T, Rosillo N, Cordova R, Cree IA, Gonzalez A (2022). Machine learning computational tools to assist the performance of systematic reviews: A mapping review. BMC Med Res Methodol.

[3] Blaizot A, Veettil SK, Saidoung P, Moreno-Garcia CF, Wiratunga N, Aceves-Martins M (2022). Using artificial intelligence methods for systematic review in health sciences: A systematic review. Res Synth Methods.

[4] dos Santos ÁO, da Silva ES, Couto LM, Reis GVL, Belo VS (2023). The use of artificial intelligence for automating or semi-automating biomedical literature analyses: A scoping review. J Biomed Inform.

[5] van Dijk SHB, Brusse-Keizer MGJ, Bucsán CC, van der Palen J, Doggen CJM, Lenferink A (2023). Artificial intelligence in systematic reviews: promising when appropriately used. BMJ Open.

[6] van de Schoot R, de Bruin J, Schram R, Zahedi P, de Boer J, Weijdema F (2021). An open source machine learning framework for efficient and transparent systematic reviews. Nat Mach Intell.

[7] Schopow N, Osterhoff G, Baur D (2023). Applications of the Natural Language Processing Tool ChatGPT in Clinical Practice: Comparative Study and Augmented Systematic Review. JMIR Med Inform.

[8] Guo E, Gupta M, Deng J, Park YJ, Paget M, Naugler C. Automated paper screening for clinical reviews using large language models. J Med Internet Res. 2023 [cited 2023 Nov 26]; Available from: http://arxiv.org/abs/2305.00844. 10.2196/48996 PMC10818236 38214966

[9] Weissenbacher D, O’Connor K, Klein A, Golder S, Flores I, Elyaderani A, et al. Text mining biomedical literature to identify extremely unbalanced data for digital epidemiology and systematic reviews: dataset and methods for a SARS-CoV-2 genomic epidemiology study. medRxiv. 2023 . 2023.07.29.23293370. [cited 2024 Jan 3]. Available from: https://www.medrxiv.org/content/https://doi.org/10.1101/2023.07.29.23293370v1 .

[10] Syriani E, David I, Kumar G (2023). Assessing the ability of ChatGPT to screen articles for systematic reviews.

[11] Alshami A, Elsayed M, Ali E, Eltoukhy AEE, Zayed T (2023). Harnessing the power of ChatGPT for automating systematic review process: methodology, case study, limitations, and future directions. Systems.

[12] Khraisha Q, Put S, Kappenberg J, Warraitch A, Hadfield K (2023). Can large language models replace humans in the systematic review process? Evaluating GPT-4’s efficacy in screening and extracting data from peer-reviewed and grey literature in multiple languages.

[13] Liang W, Zhang Y, Cao H, Wang B, Ding D, Yang X (2023). Can large language models provide useful feedback on research papers? A large-scale empirical analysis.

[14] Liu R, Shah NB (2023). ReviewerGPT? An exploratory study on using large language models for paper reviewing.

[15] Kassaye SG, Grossman Z, Balamane M, Johnston-White B, Liu C, Kumar P (2016). Transmitted HIV drug resistance is high and longstanding in Metropolitan Washington, DC. Clin Infect Dis Off Publ Infect Dis Soc Am.

[16] Tao K, Rhee SY, Chu C, Avalos A, Ahluwalia AK, Gupta RK (2023). Treatment Emergent Dolutegravir Resistance Mutations in Individuals Naïve to HIV-1 Integrase Inhibitors: A Rapid Scoping Review. Viruses.

[17] Zhang Z, Zhang A, Li M, Smola A (2022). Automatic chain of thought prompting in large language models.

[18] Kandpal N, Deng H, Roberts A, Wallace E, Raffel C (2023). Large language models struggle to learn long-tail knowledge.

