# Supplementary material for: GPT-4 performance on querying scientific publications: reproducibility, accuracy, and impact of an instruction sheet
Source: BMC Med Res Methodol. 2024 Jun 25;24:139. doi: 10.1186/s12874-024-02253-y (PMC11197181; doi:10.1186/s12874-024-02253-y)
Supplement: Supplementary file 3 — Supplementary Material 3. [file 12874_2024_2253_MOESM3_ESM.docx]

We review published papers to extract data relevant to HIV drug resistance (HIVDR). 4 main types of studies contain relevant HIVDR data: (1) In vitro passage experiments in which viruses are cultured in the presence of increasing concentrations of an antiretroviral (ARV) drug; (2) Genetic sequencing of virus isolates from patients who have or who not received ARV therapy (ART); (3) In vitro susceptibility testing (aka phenotypic testing) of viruses with drug-resistance mutations (DRMs) to determine the concentration required to inhibit the replication of these viruses by 50% (referred to as EC50 or IC50). This concentration is divided by the EC50 (or IC50) of a reference drug-susceptible virus to determine the fold-reduction in susceptibility (aka fold increase in drug resistance) of a virus to an ARV; and (4) Studies of the influence of DRMs on the virological response of patients to an ARV treatment regimen.

**HIV isolates and gene sequences:**

The genes we are interested are (1) reverse transcriptase (RT), (2) protease (PR), (3) integrase (IN or INT), and (4) capsid. HIV RT, protease, and integrase are encoded by the HIV pol gene. HIV capsid is encoded by the HIV gag gene.

We are interested in whether HIV genetic information is provided in a paper. This information can be provided in two ways: The authors can indicate whether they submitted the samples to GenBank or the authors can provide lists of amino acid mutations present in sequenced virus samples. Lists of mutations are presented as amino acid differences from a reference sequence. We are only interested in the sequences of viruses isolated by the authors or created by the authors through molecular techniques (e.g., site-directed mutagenesis).

If the sequences were submitted to GenBank, the paper may have GenBank accession numbers – which are two letters followed by six numbers. The DNA Data Bank of Japan (DDBJ) and the European Nucleotide Archive (ENA) share their data with GenBank. GenBank accession numbers contain 2 letters followed by 6 numbers. We are not interested in the sequences or GenBank accession numbers of commonly used laboratory viruses such as HXB2 or NL43.

We are collecting the following information about HIV isolates: (1) Whether they are obtained from (1) plasma samples, (2) peripheral blood mononuclear cells (PBMCs), (3) dried blood spots, (4) or whole blood. Dried blood spots and whole blood contain both plasma and PBMCs. It is important to know whether a sample contains just plasma or just PBMCs. Rarely samples are obtained from specific types of lymphocytes (which are a subset of PBMCs), lymph nodes, or cerebrospinal fluid (CSF).

Plasma HIV nucleic acids contain just RNA. PBMC HIV nucleic acids contain just DNA. HIV DNA exists in nonintegrated linear and circular episomal forms and in a form integrated into host chromosomal DNA (called proviral DNA or provirus). Nonintegrated viral DNA forms have short half-lives (less than several months). Patients whose virus levels in plasma (aka virus load) are suppressed are defined as having essentially no detectable virus in plasma (most commonly <50 HIV RNA copies per ml). In patients with virological suppression, PBMC HIV DNA consists primarily of proviral HIV DNA. The HIV reservoir is comprised of proviral DNA.

If a sample contains just PBMCs, it is essential to know whether the patient from whom the sample was obtained had attained virological suppression as a result of ARV treatment. In patients with active virus replication, DRMs detected by HIV PBMC sequencing are usually similar to those detected by plasma HIV sequencing. However, in patients whose virus loads are suppressed, DRMs detected by HIV PBMC sequencing do not reflect selection pressure from the ARV drugs the patient was receiving at the time the sample was obtained. In these patients, the DRMs detected by PBMC sequencing reflect selection pressure from previous ART regimens that were not successful at suppressing virus replication.

An increasing number of recent studies involve HIV PBMC sequencing of multiple genes in patients who are virologically suppressed. As noted above, the mutations in these sequences do not reflect ARV selection pressure. As a result, these studies usually do not contain important data about HIVDR. Rather these studies are designed to study evolution of the HIV reservoir.

Virus samples may be sequenced directly after PCR (or RT-PCR) is performed – often referred to as direct PCR or population-based sequencing. Virus samples may also be cloned prior to sequencing. Molecular cloning involves ligating the virus genes of interest into a plasmid and transforming bacteria with the plasmid. Studies in which HIV was cloned prior to sequencing usually contain several additional sentences describing this process. Another method of cloning, called single genome sequencing (SGS) or single genome amplification (SGA) involves limiting dilution analysis of reverse-transcribed virus RNA (also known as complementary DNA or cDNA).

Sequencing is performed using two main types of methods. Sanger (aka dideoxyterminator or dideoxynucleotide) sequencing was the only method used prior to 2007. Companies involved in Sanger sequencing included ABI (ViroSeq) and Visible Genetics (TruGene). Next-generation sequencing (NGS) is being increasingly used but is still less common than Sanger sequencing. The main NGS methods involve using Illumina, Nanopore, Ion Torrent, and PacBio. Genotypic resistance testing refers to sequencing of the genes that are the targets of ART. This usually refers to the RT, protease, and integrase genes.

It is possible for multiple sequences to be reported for the same patient. These could be sequences obtained at different times such as sequences obtained before and after an ART regimen. These could also represent multiple sequences from the same virus isolate that were determined using molecular or limiting dilution cloning. If NGS was performed, the authors may report the number of sequence reads obtained for the sequenced sample. If NGS was performed, the authors may report the proportion of reads containing a mutation.

There two HIV species HIV-1 and HIV-2. HIV-1 is much more prevalent. There have been four recognized HIV-1 cross species transmissions from primates to humans: groups M, N, O, and P. Group M is responsible for the global HIV-1 pandemic while groups N, O, and P viruses are extremely rare. During its spread among humans, group M HIV-1 has evolved into multiple subtypes and circulating recombinant forms (CRFs).

Additional information of importance from studies of HIVDR include the regions and countries where samples were obtained for sequencing, the years the samples were obtained, and whether the persons from whom the samples were obtained were children or adults. Infants and children less than 15 years old are likely to have been infected perinatally.

**HIV treatments:**

There are about 30 FDA-approved ARVs. The most commonly used are: (1) the nucleoside RT inhibitors (NRTIs), including the cytosine analogs lamivudine (3TC) and emtricitabine (FTC), the tenofovir prodrugs tenofovir disoproxil fumarate (TDF) and tenofovir alafenamide (TAF), and zidovudine (AZT or ZDV) and abacavir (ABC); (2) the nonnucleoside RT inhibitors (NNRTIs), including nevirapine (NVP), efavirenz (EFV), rilpivirine (RPV), etravirine (ETR), and doravirine (DOR); (3) the pharmacologically boosted protease inhibitors (PIs), including ritonavir-boosted lopinavir (LPV; LPV/r) and ritonavir- or cobicistat boosted atazanavir (ATV; ATV/r or ATV/c) and darunavir (DRV; DRV/r or DRV/c); and (4) the first-generation integrase strand transfer inhibitors (INSTIs) raltegravir and elvitegravir, and the second-generation INSTIs dolutegravir (DTG), bictegravir (BIC), and cabotegravir (CAB). There are 4 FDA approved entry inhibitors. There is 1 FDA approved capsid (CA) inhibitor (CAI)– lenacapavir.

The most commonly used ART regimens include 2 NRTIs plus either an NNRTI, a PI, or an INSTI. Since about 2010, the most commonly used NRTIs have included either 3TC or FTC (sometimes collectively referred to as XTC) and TDF or TAF (sometimes collectively referred to as TFV or TXF). AZT and ABC are occasionally used with 3TC. Prior to 2010, another NRTI stavudine (D4T) was often used with 3TC in low- and middle-income countries (LMICs). In the early 2000s, most first-line ART regimens in both LMICs and upper-Income countries (UICs) consisted of 2 NRTIs plus an NNRTI, most commonly NVP or EFV. For the past 5 years, 2 NRTIs plus the INSTI DTG have been recommended for first-line ART in LMICs. For the past 10 years, 2 NRTIs plus an INSTI have been recommended for first-line ART in UICs. Currently, the following INSTIs are recommended for first-line ART in UICs: DTG + 2 NRTIs, BIC + 2 NRTIs, and DTG + 3TC.

Important information from a paper regarding treatment would include (1) How many patients received ART (ART-experienced) and how many had not (i.e., were ART-naïve); (2) How many ART-experienced patients received an ARV belonging to specific ARV classes; (3) Which drugs in each drug class were received among ART-experienced patients; and (4) How many ART regimens were received by a patient (i.e., the patient’s ART history). It is important to know whether a patient received just a single ARV in a drug class because in that case, it is likely that the DRMs associated with the drug class were selected by the ARV the patient had received. It is important to know whether the ART history of a patient was complete. If the ART history is not complete, it is useful to know which ARVs were definitely not received. For example, it may be known that a patient received an INSTI but did not receive a second-generation INSTI. Sometimes information will only be presented about the type of regimen received. For example, a patient in a LMIC who received a first-line regimen, would have received 2 NRTIs and an NNRTI. The NRTIs would include either 3TC or FTC and either TFV or AZT (less likely D4T or ABC). The NNRTIs would include NVP or EFV. The ART history from a patient in a LMIC receiving a second-line regimen would have received a first-line regimen followed by a second-line regimen containing a PI (most commonly LPV/r) plus 2 NRTIs.

Patients who are newly diagnosed are by definition ART-naïve. Patients with transmitted drug resistance (TDR) had DRMs before they were treated. Pre-treatment drug resistance (PDR) usually refers to patients with TDR.

In many studies, there are patients with different ART histories. The information linking a patient identifier to their ART history may be found in a table or in the supplementary material of a paper. These data may be necessary to link the mutations in an HIV sequence to the ART history of the patient from whom the sequence was obtained.

We distinguish studies of HIVDR from studies that use HIV pol sequences to study transmission patterns (also referred to as molecular epidemiology or phylogenetic studies). Molecular epidemiology have little granular information linking HIV sequences to the ART history of the person from whom sequences are obtained. Instead, molecular epidemiological studies use the similarity between sequences to identify clusters of sequences from HIV-infected patients sharing similar epidemiological characteristics.

**HIV drug susceptibility (phenotypic) data:**

Phenotypic testing is usually performed on site-directed mutants, virus isolates that developed mutations during in vitro passage experiments, or on clinical viruses samples obtained directly from a patient sample. If phenotypic testing was performed, there are usually one or more sentences describing the phenotypic test method. The most common method is the PhenoSense assay (which can also be referred to as PhenoSenseGT).

Many studies report that patients had one of the following levels of resistance: potential low-level, low-level, intermediate, and high-level. Unless a fold reduction in resistance is reported, these levels are determined by an interpretation program applied to a list of mutations in a sequence.

Patient population:

It is often useful to know if the patients in a study were part of a clinical trial because in such studies the ART histories of the patients are more likely to be less heterogeneous.

A study may describe a large number of patient. We are primarily interested in those who underwent genotypic resistance testing.
