# Supplementary material for: GPT-4 performance on querying scientific publications: reproducibility, accuracy, and impact of an instruction sheet
Source: BMC Med Res Methodol. 2024 Jun 25;24:139. doi: 10.1186/s12874-024-02253-y (PMC11197181; doi:10.1186/s12874-024-02253-y)
Supplement: Supplementary file 5 — Supplementary Material 5. [file 12874_2024_2253_MOESM5_ESM.docx]

**__init__.py**

from .option import select_chat_mode

from .chat_auto import chat_auto

from .chat_repl import chat_repl

**# select_chat_mode() asks the command-line user to supply all parameters for building**

**# the open AI prompt. The parameters include: list of papers (by PMID), list of**

**# questions (by ID), configuration (e.g., w/wo instruction sheet).**

**# chat_auto will call the main function that build and run the prompt.**

def chat():

chat_context = select_chat_mode()

chat_func = chat_context['chat_func']

del chat_context['chat_func']

if not chat_context['auto_mode?']:

chat_repl(chat_context, chat_func)

else:

chat_auto(chat_context, chat_func)

**option.py**

from prompt_toolkit.shortcuts import yes_no_dialog

from prompt_toolkit.shortcuts import input_dialog

from src.exceptions import check_selection

from src.preset import MODEL_RATE_LIMITS

from src.preset import DEFAULT_OPTIONS

from src.select_content.question import select_question

from src.select_content.question import select_question_set

from src.select_content.prompt_template import select_question_template

from src.select_content.llm_model import select_model

from src.select_content.paper import select_paper_content

from src.select_content.test_set import select_test_set

from src.select_content.prompt_template import load_prompt_template

from src.select_content.instruction import select_instruction

from .chat_mode.one_q_all_content import one_q_all_content

from .chat_mode.multi_q_all_content import multi_q_all_content

from .chat_mode.one_q_embedding import one_q_embedding

from src.apis.embedding import get_token_length

from dataclasses import dataclass

from types import FunctionType

def select_chat_mode():

**# Selection which Large language model API to use**

model = select_model()

**# Choose auto query mode or manual query mode**

auto_mode = choose_auto_mode()

**# Choose paper test set**

test_set = select_test_set()

**# When in manual query model, select papers, select prompt template**

**# The question will be prompted to input later.**

if not auto_mode:

papers = select_paper_content(test_set, only_one=True)

questions = {}

question_template = select_question_template(multi_questions=False)

question_mode = 'one_question_per_req'

paper_template = load_prompt_template('paper_content')

model_limit = MODEL_RATE_LIMITS[model]

reserved_token = 300

req_token_limit = model_limit['MAX_TOKENS'] - reserved_token

return {

'model': model,

'questions': questions,

'papers': papers,

'question_template': question_template,

'paper_template': paper_template,

'auto_mode?': auto_mode,

'one_question_per_req?': True,

'embedding?': False,

'cheatsheet?': False,

'cheatsheet': '',

'chat_func': None,

'chat_mode': 'manual chat',

'reserved_token': reserved_token,

'req_token_limit': req_token_limit,

}

papers = select_paper_content(test_set, all_option=True)

question_set = select_question_set()

questions = select_question(question_set, all_option=True)

**# Select w/wo embedding**

w_embedding = choose_embedding_mode()

embedding_mode = 'embedding' if w_embedding else 'all_content'

if w_embedding:

question_mode = 'one_question_per_req'

question_template = select_question_template(multi_questions=False)

multi_question = False

else:

multi_question = choose_multi_ques_mode()

question_mode = (

'multi_questions'

if multi_question

else 'one_question_per_req')

if not multi_question:

question_template = select_question_template(multi_questions=False)

else:

question_template = select_question_template(multi_questions=True)

**# Select w/wo cheatsheet**

w_cheatsheet = choose_cheatsheet_mode()

if w_cheatsheet:

instruction_template = load_prompt_template('instruction')

instruction = select_instruction()

cheatsheet = instruction_template + instruction

else:

cheatsheet = ''

**# make sure the chat mode is allowed**

if (question_mode, embedding_mode) not in CHAT_MODE_LIST:

raise Exception(f'{question_mode}, {embedding_mode} not supported')

**# Get main chat function by user preference**

chat_func = CHAT_MODE_LIST[(question_mode, embedding_mode)]

cheatsheet_mode = 'with_cheatsheet' if w_cheatsheet else 'wo_cheatsheet'

paper_template = load_prompt_template('paper_content')

**# Calculate the remain tokens can be used in one query**

model_limit = MODEL_RATE_LIMITS[model]

reserved_token = 300 if not multi_question else 1000

req_token_limit = model_limit['MAX_TOKENS'] - reserved_token

if w_cheatsheet:

req_token_limit -= get_token_length(cheatsheet)

run_number = input_dialog(

title='Please enter run number',

text='Please enter run number:').run()

if not run_number.isdigit():

run_number = 1

else:

run_number = int(run_number)

return {

'model': model,

'questions': questions,

'papers': papers,

'question_template': question_template,

'paper_template': paper_template,

'auto_mode?': auto_mode,

'one_question_per_req?': not multi_question,

'embedding?': embedding_mode,

'cheatsheet?': w_cheatsheet,

'cheatsheet': cheatsheet if w_cheatsheet else '',

'remove_sent?': False,

'append_sent?': False,

'chat_func': chat_func,

'chat_mode': ', '.join([

question_mode, embedding_mode, cheatsheet_mode]),

'reserved_token': reserved_token,

'req_token_limit': req_token_limit,

'run_number': run_number,

}

**# All allowed chat mode and the corresponding functions.**

CHAT_MODE_LIST = {

('one_question_per_req', 'all_content'): one_q_all_content,

('multi_questions', 'all_content'): multi_q_all_content,

('one_question_per_req', 'embedding'): one_q_embedding,}

**# Command line interactive function**

@check_selection()

def choose_auto_mode(

title="Auto or REPL mode", desc="Automatically chat the paper?"):

if 'auto_mode' in DEFAULT_OPTIONS:

return DEFAULT_OPTIONS['auto_mode']

result = yes_no_dialog(

title=title,

text=desc,

).run()

return result

@check_selection()

def choose_remove_sentence(

title="Remove sentence from cheatsheet",

desc="Remove sentence from cheatsheet?"):

if 'replace_sent?' in DEFAULT_OPTIONS:

return DEFAULT_OPTIONS['replace_sent?']

result = yes_no_dialog(

title=title,

text=desc,

).run()

return result

@check_selection()

def choose_append_sentence(

title="Append sentence to cheatsheet",

desc="Append sentence to cheatsheet?"):

if 'append_sent?' in DEFAULT_OPTIONS:

return DEFAULT_OPTIONS['append_sent?']

result = yes_no_dialog(

title=title,

text=desc,

).run()

return result

@check_selection()

def choose_multi_ques_mode(

title="Question mode", desc="Multiple questions per request?"):

if 'multiple_questions?' in DEFAULT_OPTIONS:

return DEFAULT_OPTIONS.get('multiple_questions?')

result = yes_no_dialog(

title=title,

text=desc,

).run()

return result

@check_selection()

def choose_embedding_mode(

title="Using embedding method?",

desc=""):

if 'embedding?' in DEFAULT_OPTIONS:

return DEFAULT_OPTIONS.get('embedding?')

result = yes_no_dialog(

title=title,

text=title,

).run()

return result

@check_selection()

def choose_cheatsheet_mode(

title="Cheatsheet mode",

desc="Using cheatsheet?"):

if 'cheatsheet?' in DEFAULT_OPTIONS:

return DEFAULT_OPTIONS.get('cheatsheet?')

result = yes_no_dialog(

title=title,

text=desc,

).run()

return result

**chat_auto.py**

from copy import deepcopy

**# Called by __init__.py. Provides the object chat_context and the function chat_func**

**# Each paper is then dispatched to chat_func**

def chat_auto(chat_context, chat_func):

papers = chat_context['papers']

del chat_context['papers']

for paper in papers:

chat_func(paper, deepcopy(chat_context))

**map_reduce.py**

from src.apis.chat_api import chat_ai

from src.apis.embedding import get_token_length

from .filter_question import get_unanswered

from src.checksum import get_md5

from openai.error import Timeout

import warnings

**# Builds the prompt and calls the GPT-4 API**

def try_map_reduce(questions, chat_context):

segment_content(questions, chat_context)

resp_list = []

for i in chat_context['batches']:

resp_list.append(

process_one_batch(questions, i, chat_context))

return resp_list

**# Check the token length for the complete prompt that won’t above the token length**

**# limit of GPT-4 API**

def segment_content(questions, chat_context):

prompts = []

if chat_context['cheatsheet?']:

prompts.append(chat_context['cheatsheet'])

prompts.append(

chat_context['question_template'].format(

question=get_question_list_str(questions))

)

doc_parts = chat_context['doc_parts']

[

i.update({'token_length': get_token_length(i['all_content'])})

for i in doc_parts

]

result = []

one_batch = []

for i in doc_parts:

new_content = i['all_content']

parts = '\n'.join(one_batch + [new_content])

p_prompt = chat_context['paper_template'].format(

paper_content=parts)

prompt = '\n'.join(prompts + [p_prompt])

prompt_length = get_token_length(prompt, chat_context['model'])

if prompt_length < chat_context['req_token_limit']:

one_batch.append(new_content)

else:

one_batch_content = '\n'.join(one_batch)

result.append({

'batch_content': one_batch_content,

'content_length': get_token_length(one_batch_content),

'num_parts': len(one_batch),

})

one_batch = [new_content]

if one_batch:

one_batch_content = '\n'.join(one_batch)

result.append({

'batch_content': one_batch_content,

'content_length': get_token_length(one_batch_content),

'num_parts': len(one_batch),

})

chat_context['batches'] = result

return result

**# Query the prompt using multiple question mode**

def process_one_batch(questions, part, chat_context):

if len(questions) > 1:

try:

process_multi_questions(questions, part, chat_context)

except Timeout:

for qid, q in questions.items():

process_one_question({qid: q}, part, chat_context)

else:

process_one_question(questions, part, chat_context)

**# For asking one question for one paper.**

**# This is a fallback when submitting multiple questions times out.**

def process_one_question(unanswered, part, chat_context):

unanswered = get_unanswered(

unanswered, part['batch_content'],

chat_context['chat_history'],

chat_context['run_number'])

if not unanswered:

return

prompts = []

if chat_context['cheatsheet?']:

prompts.append(chat_context['cheatsheet'])

prompts.append(

chat_context['question_template'].format(

question=get_question_list_str(unanswered))

)

prompts.append(

chat_context['paper_template'].format(

paper_content=part['batch_content']

)

)

prompt = '\n'.join(prompts)

resp = chat_ai(prompt, chat_context['model'])

if resp['answer'] is None:

resp['answer'] = 'AI answers NA'

resp['question'] = list(unanswered.values())[0]

resp['question_id'] = list(unanswered.keys())[0]

resp['md5'] = get_md5(part['batch_content'])

resp['#batches'] = len(chat_context['batches'])

resp['run_number'] = chat_context['run_number']

chat_context['chat_history'].log(

resp,

{resp['question_id']: resp['question']})

**# Main code for submitting multiple questions**

**# When some questions were not answered, the function will try to query the remain**

**# questions.**

def process_multi_questions(unanswered, part, chat_context, retry_time=10):

unanswered = get_unanswered(

unanswered, part['batch_content'],

chat_context['chat_history'],

chat_context['run_number'])

unanswered = get_question_prompt(unanswered, chat_context)

while len(unanswered) > 0 and retry_time:

process_questions(part, unanswered, chat_context)

unanswered = get_unanswered(

unanswered, part['batch_content'],

chat_context['chat_history'],

chat_context['run_number'])

retry_time -= 1

**# Build a list of question in the prompt**

def get_question_list_str(questions):

return "\n".join([

f"{k}. {v}"

for k, v in questions.items()

])

**# Build the main prompt and submit the prompt to GPT-4 API**

**# Then process and store the answers.**

def process_questions(part, questions, chat_context):

prompts = []

if chat_context['cheatsheet?']:

prompts.append(chat_context['cheatsheet'])

question_str = get_question_list_str(questions)

prompts.append(

chat_context['question_template'].format(

question=question_str)

)

prompts.append(

chat_context['paper_template'].format(

paper_content=part['batch_content'])

)

prompt = '\n'.join(prompts)

resp = chat_ai(prompt, chat_context['model'])

if resp['answer'] is None:

raise KeyError('content')

resp['md5'] = get_md5(part['batch_content'])

resp['#batches'] = len(chat_context['batches'])

resp['run_number'] = chat_context['run_number']

chat_context['chat_history'].log(resp, questions)

return resp

**multi_q_all_content.py**

from src.doc_format.md import split_md_section

from ..map_reduce import try_map_reduce

from ..chat_history import ChatHistory

**# Query in multiple question mode**

def multi_q_all_content(paper_file_path, chat_context, num_batch=60):

chat_history = ChatHistory(paper_file_path, chat_context)

chat_context['chat_history'] = chat_history

chat_context['doc_parts'] = split_md_section(paper_file_path)

**# if the question need to be splited into batches, the group the question in**

**# batches, fallback to 1 if no num_batch is indicated.**

while num_batch >= 1:

batches = get_batches(chat_context['questions'], num_batch)

try:

try_batches(batches, chat_context)

break

except KeyError as e:

if num_batch <= 1:

raise e

num_batch = 1

chat_history.dump_log()

**# Call the main function try_map_reduce**

def try_batches(batches, chat_context):

for one_batch in batches:

try_map_reduce(dict(one_batch), chat_context)

def get_batches(questions, batch):

batches = []

questions = list(questions.items())

num_batch = len(questions) // batch + (1 if len(questions) % batch else 0)

for i in range(num_batch):

one_batch = questions[i * batch: (i + 1) * batch]

batches.append(dict(one_batch))

return batches

**one_q_all_content.py**

from src.doc_format.md import split_md_section

from ..chat_history import ChatHistory

from ..map_reduce import try_map_reduce

def one_q_all_content(paper_file_path, chat_context):

chat_history = ChatHistory(paper_file_path, chat_context)

chat_context['chat_history'] = chat_history

chat_context['doc_parts'] = split_md_section(paper_file_path)

for qid, q in chat_context['questions'].items():

try_map_reduce({qid: q}, chat_context)

chat_history.dump_log()

**chat_repl.py**

from prompt_toolkit.shortcuts import input_dialog

from prompt_toolkit.shortcuts import message_dialog

from prompt_toolkit.shortcuts import yes_no_dialog

from copy import deepcopy

**# Manually provide customized or updated single question to query GPT-4 API**

def chat_repl(chat_context, chat_func):

paper = chat_context['papers'][0]

qid = 1000 **# manual question id**

while True:

question = get_question()

if (question.lower().strip() in ('quit', '')):

break

qid += 1

chat_context['questions'] = {qid: question}

answer = chat_func(paper, deepcopy(chat_context))

show_answer(answer)

_continue = yes_no_dialog(

title='Continue?',

text='').run()

if not _continue:

break

def get_question(cmd=True):

if cmd:

question = input('\nPlease enter your question: ')

else:

question = input_dialog(

title='Please enter your question',

text='',

default=''

).run()

return question

def show_answer(answer, cmd=True):

if cmd:

print(f"Answer:\n{answer}")

else:

message_dialog(

title='Answer',

text=answer).run()

**chat_api.py**

from datetime import datetime

import openai

import time

from src.select_content.prompt_template import load_prompt_template

from .rate_limit import RateLimit

from src.apis.embedding import get_token_length

from .openai_api import chat_openai

from .azure_api import chat_azure

def retry_api(func):

def wrapper(*args, **kwargs):

retry_times = 3

start_time = datetime.now()

while retry_times > 0:

try:

answer = func(*args, **kwargs)

retry_times = 0

except openai.error.RateLimitError as e:

time.sleep(90)

retry_times -= 1

except openai.error.APIError as e:

time.sleep(90)

except openai.error.ServiceUnavailableError as e:

time.sleep(90)

except openai.error.Timeout as e:

time.sleep(90)

seconds = (datetime.now() - start_time).seconds

answer['seconds'] = seconds

return answer

return wrapper

rate_limit = RateLimit()

@retry_api

def chat_ai(prompt, model="gpt-3.5-turbo", temperature=0):

rate_limit = RateLimit()

if rate_limit.chech_hit_context_length(prompt, model):

return {

'answer': 'Prompt too long'

}

if rate_limit.check_hit_limit(prompt, model):

time.sleep(90)

messages = [

{"role": "system", "content": load_prompt_template('system')},

{"role": "user", "content": prompt}]

# response = chat_openai(model, messages, temperature)

response = chat_azure(model, messages, temperature)

answer = response.choices[0].message.get("content")

result = {

'answer': answer,

'completion_tokens': response.usage.completion_tokens,

'prompt_tokens': response.usage.prompt_tokens,

'total_tokens': response.usage.total_tokens,

}

**openai_api.py**

import openai

import os

def chat_openai(model, messages, temperature=0):

openai.api_key = os.getenv('OPENAI_API_KEY')

response = openai.ChatCompletion.create(

model=model,

messages=messages,

temperature=temperature

)

return response

**azure_api.py**

import openai

import os

def chat_azure(model, messages, temperature=0):

openai.api_key = os.getenv('AZURE_OPEN_AI_API_KEY')

openai.api_base = os.getenv('AZURE_OPEN_AI_API_ENDPOINT')

openai.api_type = os.getenv('AZURE_OPEN_AI_API_TYPE')

openai.api_version = os.getenv('AZURE_OPEN_AI_API_VERSION')

if model == 'gpt-4-32k':

engine = os.getenv('AZURE_GPT4_32K_ID')

else:

raise Exception(f"Not supported model {model}")

response = openai.ChatCompletion.create(

engine=engine,

messages=messages,

temperature=temperature

)

return response
