# Supplementary material for: GPT-4 performance on querying scientific publications: reproducibility, accuracy, and impact of an instruction sheet
Source: BMC Med Res Methodol. 2024 Jun 25;24:139. doi: 10.1186/s12874-024-02253-y (PMC11197181; doi:10.1186/s12874-024-02253-y)
Supplement: Supplementary file 6 — Supplementary Material 6. [file 12874_2024_2253_MOESM6_ESM.docx]

**Appendix 6: GPT-4 Responses to Questions Designed to Determine the Extent of its Pre-Existing Knowledge of Information Provided in the Instruction Sheet**

**Question 1 How are the results of HIV-1 in vitro susceptibility testing reported?**

Instruction sheet text with relevant information

In vitro susceptibility testing (aka phenotypic testing) of viruses with drug-resistance mutations (DRMs) to determine the concentration required to inhibit the replication of these viruses by 50% (referred to as EC50 or IC50). This concentration is divided by the EC50 (or IC50) of a reference drug-susceptible virus to determine the fold-reduction in susceptibility (aka fold increase in drug resistance) of a virus to an ARV.

GPT-4 answer

The results of HIV-1 in vitro susceptibility testing are typically reported in terms of drug concentration required to inhibit viral replication by a certain percentage (usually 50% or 90%) compared to a control without the drug. These measurements are referred to as the IC50 (inhibitory concentration 50%) and IC90 (inhibitory concentration 90%). Here's a more detailed breakdown:

Inhibitory Concentration 50% (IC50): This value represents the concentration of a drug needed to inhibit viral replication by 50% under specific laboratory conditions. It is a common measure used to evaluate the potency of an antiretroviral drug against the virus. A lower IC50 value indicates greater potency, meaning less drug is required to achieve the desired inhibitory effect.

Inhibitory Concentration 90% (IC90): Similarly, the IC90 value indicates the concentration of the drug required to inhibit 90% of viral replication in vitro. This value is less commonly reported but provides additional information about the drug's effectiveness at higher concentrations.

Fold Change: The results may also be expressed as a fold change in IC50 (or IC90) compared to a reference wild-type HIV-1 strain. This measures how much more (or less) drug is needed to inhibit a particular HIV-1 strain compared to the reference strain. A fold change of 1 indicates no change in susceptibility, while a fold change greater than 1 indicates reduced susceptibility (resistance), and a fold change less than 1 indicates increased susceptibility.

Resistance Interpretation: Some reports may include a qualitative interpretation of these quantitative results, categorizing the HIV-1 strain as "sensitive," "intermediate," or "resistant" to the tested antiretroviral drug based on predefined thresholds. These interpretations help clinicians understand the clinical relevance of the test results and make informed decisions about antiretroviral therapy (ART).

The specific methodologies and cutoff values used can vary between different laboratories and testing platforms. It's essential for healthcare providers to understand the context and limitations of these tests to appropriately interpret the results for clinical decision-making.

Assessment

GPT-4 has an excellent understanding of HIV-1 in vitro susceptibility testing.

**Question 2 How is it possible to know whether a paper is reporting the results of HIV-1 genotypic resistance testing?**

Instruction sheet text with relevant information

We are interested in whether HIV genetic information is provided in a paper. This information can be provided in two ways: The authors can indicate whether they submitted the samples to GenBank or the authors can provide lists of amino acid mutations present in sequenced virus samples. Lists of mutations are presented as amino acid differences from a reference sequence. We are only interested in the sequences of viruses isolated by the authors or created by the authors through molecular techniques (e.g., site-directed mutagenesis).

GPT-4 answer

Determining whether a paper is reporting the results of HIV-1 genotypic resistance testing involves identifying specific indicators within the text. Genotypic resistance testing is a method used to identify mutations in the HIV-1 genome that confer resistance to antiretroviral drugs. Here are some key features and terms to look for that can indicate a paper is reporting the results of HIV-1 genotypic resistance testing:

Terminology and Phrases: Look for specific terms such as "genotypic resistance testing," "genotypic analysis," "HIV-1 mutations," "drug resistance mutations (DRMs)," or "resistance-associated mutations (RAMs)." These terms suggest the paper is focused on analyzing the genetic sequence of the virus to identify mutations related to drug resistance.

Methodology: The paper should describe the method used to sequence the HIV-1 genome and identify mutations. This might include details about the type of sequencing technology used (e.g., Sanger sequencing, next-generation sequencing), the specific genomic regions analyzed (e.g., the reverse transcriptase or protease genes), and the bioinformatics tools or databases used to interpret the sequence data.

Results Section: In the results section, papers reporting genotypic resistance testing will typically list specific mutations identified in the HIV-1 genome and may correlate these mutations with resistance to specific antiretroviral drugs or drug classes. The presence of tables or figures summarizing the frequency of specific mutations or patterns of resistance within the study population is another strong indicator.

Discussion and Interpretation: The discussion section should provide an interpretation of the significance of the identified mutations in the context of HIV treatment and drug resistance. This might include discussions on the impact of specific mutations on drug efficacy, potential treatment options given the resistance profile, and recommendations for managing patients with drug-resistant HIV-1 strains.

References to Guidelines or Databases: Papers focused on genotypic resistance testing often reference guidelines for interpreting genotypic resistance data (e.g., those from the International Antiviral Society-USA or the European AIDS Clinical Society) or databases that catalog known resistance mutations (e.g., the Stanford HIV Drug Resistance Database).

By looking for these features and terms, you can confidently identify papers that are reporting the results of HIV-1 genotypic resistance testing.

Assessment

GPT-4 has the knowledge of genotypic resistance testing.

**Question 3 How can we determine if the genetic sequences in a paper about HIV drug resistance have been submitted to GenBank?**

Instruction sheet text with relevant information

If the sequences were submitted to GenBank, the paper may have GenBank accession numbers – which are two letters followed by six numbers. The DNA Data Bank of Japan (DDBJ) and the European Nucleotide Archive (ENA) share their data with GenBank. GenBank accession numbers contain 2 letters followed by 6 numbers.

GPT-4 answer

Determining if the genetic sequences from a paper about HIV drug resistance have been submitted to GenBank involves a few key steps or indicators to look for within the paper itself or through external databases. GenBank is a publicly accessible database of genetic sequences that researchers use to share their findings with the broader scientific community. Here’s how you can identify whether sequences from a specific study have been submitted to GenBank:

Check the Paper for Accession Numbers:

Within the Text: Researchers often include GenBank accession numbers in the paper, either within the text when discussing the sequences, in the figure or table captions where sequences are presented, or in a supplementary information section. Accession numbers are unique identifiers assigned by GenBank to each sequence submission.

Materials and Methods Section: The section describing the methodology might mention that sequences have been submitted to GenBank and may provide the accession numbers or a range of numbers.

Look for Supplementary Materials:

Papers often have supplementary materials that include detailed data not presented in the main text. These materials might list the GenBank accession numbers for all sequences used or generated in the study.

Search GenBank Directly:

You can search the GenBank database directly by using keywords from the paper, such as the authors' names, specific genes studied, or the study's title. If the sequences have been submitted, the search should return entries with matching details.

Visit the NCBI website and use the search bar to enter your query, or navigate to the Nucleotide database to perform a more focused search.

Contact the Authors:

If the paper does not explicitly mention GenBank accession numbers and you cannot find the sequences through a database search, consider contacting the corresponding author for clarification. Authors are usually willing to share this information upon request.

Publication Guidelines and Journal Policies:

Some journals require authors to submit all relevant genetic sequences to a public database like GenBank as a condition of publication. Review the journal’s author guidelines or the specific article’s declaration or ethics statement sections to see if there is a mention of such a requirement.

By following these steps, you should be able to determine whether the genetic sequences discussed in a paper about HIV drug resistance have been submitted to GenBank.

Assessment

GPT-4 has knowledge about finding if sequences were submitted to GenBank.

**Question 4 What is the difference between sequences from HIV plasma samples and sequences from PBMC samples?**

Instruction sheet text with relevant information

Plasma HIV nucleic acids contain just RNA. PBMC HIV nucleic acids contain just DNA. HIV DNA exists in nonintegrated linear and circular episomal forms and in a form integrated into host chromosomal DNA (called proviral DNA or provirus). Nonintegrated viral DNA forms have short half-lives (less than several months). Patients whose virus levels in plasma (aka virus load) are suppressed are defined as having essentially no detectable virus in plasma (most commonly <50 HIV RNA copies per ml). In patients with virological suppression, PBMC HIV DNA consists primarily of proviral HIV DNA. The HIV reservoir is comprised of proviral DNA. If a sample contains just PBMCs, it is essential to know whether the patient from whom the sample was obtained had attained virological suppression as a result of ARV treatment. In patients with active virus replication, DRMs detected by HIV PBMC sequencing are usually similar to those detected by plasma HIV sequencing. However, in patients whose virus loads are suppressed, DRMs detected by HIV PBMC sequencing do not reflect selection pressure from the ARV drugs the patient was receiving at the time the sample was obtained. In these patients, the DRMs detected by PBMC sequencing reflect selection pressure from previous ART regimens that were not successful at suppressing virus replication.

GPT-4 answer

The difference between sequences from HIV plasma samples and sequences from PBMC (Peripheral Blood Mononuclear Cells) samples primarily lies in the type of material being analyzed and what these materials represent in the context of HIV infection.

HIV Plasma Samples:

Plasma samples are obtained from the blood, with cells removed, leaving behind the clear liquid portion. This liquid contains free-floating HIV RNA, representing the virus that is currently circulating in the bloodstream.

Sequences obtained from plasma samples primarily reflect the current, active viral population in an individual's blood. This can be particularly relevant for understanding the viral load and the active replication of HIV.

Analysis of sequences from plasma can be crucial for monitoring treatment effectiveness, detecting drug resistance, and understanding the dynamics of HIV evolution and transmission.

PBMC Samples:

PBMCs are a type of blood cell that includes lymphocytes (such as T cells and B cells) and monocytes, which are essential components of the immune system. These cells can be infected by HIV, serving as a reservoir for the virus.

Sequences obtained from PBMC samples can provide information about the integrated proviral DNA within these cells. This represents not just the currently active virus but also latent and archived versions of the virus that may not be circulating in the plasma.

Analysis of sequences from PBMCs can offer insights into the long-term persistence of HIV, the reservoir of HIV within an individual, and the genetic diversity of the virus over time. This is especially relevant for understanding the challenges in achieving a cure for HIV, as these reservoirs can reactivate infection if treatment is interrupted.

Key Differences:

Material Analyzed: Plasma samples focus on free viral RNA in the bloodstream, while PBMC samples analyze viral DNA integrated into the genome of blood cells.

Representation of Viral Population: Plasma sequences represent the actively replicating virus, while PBMC sequences represent both active and latent virus populations.

Clinical and Research Implications: Plasma sequences are critical for monitoring infection dynamics and treatment response. In contrast, PBMC sequences are vital for understanding the persistence and reservoirs of HIV, with significant implications for cure research.

Each type of sample offers unique and complementary insights into the state of HIV infection, its dynamics, and the challenges in managing and potentially eradicating the virus.

Assessment

GPT-4 has knowledge about the difference between plasma and PBMC samples.

**Question 5 What are different methods of sequencing the HIV virus?**

Instruction sheet text with relevant information

Sequencing is performed using two main types of methods. Sanger (aka dideoxyterminator or dideoxynucleotide) sequencing was the only method used prior to 2007. Companies involved in Sanger sequencing included ABI (ViroSeq) and Visible Genetics (TruGene). Next-generation sequencing (NGS) is being increasingly used but is still less common than Sanger sequencing. The main NGS methods involve using Illumina, Nanopore, Ion Torrent, and PacBio.

GPT-4 answer

Sequencing the HIV virus involves determining the precise order of nucleotides (adenine, guanine, cytosine, and thymine) in its RNA genome. This is critical for understanding the virus's structure, how it mutates, and how it can be targeted by treatments. There are several methods used to sequence HIV, each with its own strengths and applications:

Sanger Sequencing: This classic method, also known as the chain termination method, has been used since the 1970s. It involves synthesizing a complementary DNA (cDNA) strand to the HIV RNA, then terminating the synthesis process at random points using dideoxynucleotides. The resulting fragments are separated by size using gel electrophoresis, allowing the sequence to be read. It's highly accurate for short sequences but less effective for sequencing entire genomes or detecting minor variants within a population.

Next-Generation Sequencing (NGS): This encompasses several technologies that allow for the simultaneous sequencing of millions of DNA fragments. For HIV, this often involves reverse transcribing the virus's RNA into DNA, fragmenting the DNA, and then sequencing these fragments in parallel. NGS can provide a deep understanding of the genetic diversity within viral populations, including minority variants that might confer drug resistance. However, it can be more complex and expensive than some other methods.

Illumina sequencing is a widely used NGS method that sequences DNA by synthesizing the complementary strand and incorporating fluorescently labeled nucleotides. The fluorescence is detected to identify the nucleotide added at each step.

Pyrosequencing (454 sequencing) works by adding one nucleotide at a time across all the DNA templates and detecting the light emitted during the incorporation of the nucleotide into the new DNA strand.

Ion Torrent sequencing uses a semiconductor chip to detect hydrogen ions released during the polymerization of DNA, allowing nucleotide identification.

Third-Generation Sequencing (Long-Read Sequencing): Technologies like Pacific Biosciences (PacBio) and Oxford Nanopore allow for the sequencing of much longer DNA fragments than NGS, in some cases up to hundreds of thousands of nucleotides without interruption. This is particularly useful for resolving complex regions of the HIV genome or for understanding the structure of integrated HIV DNA within the human genome. These methods offer the advantage of longer reads, which can simplify genome assembly and the identification of structural variations but might have higher error rates compared to NGS.

Each method has its own advantages and limitations, including cost, accuracy, read length, and the ability to detect rare variants. The choice of sequencing method depends on the specific goals of the research or diagnostic test, such as studying the evolution and transmission of the virus, monitoring drug resistance, or developing vaccines.

Assessment

GPT-4 has knowledge about common HIV-1 sequencing methods.

**Question 6 Explain all the HIV-1 and HIV-2 groups and prevalence of each group.**

Instruction sheet text with relevant information

There two HIV species HIV-1 and HIV-2. HIV-1 is much more prevalent. There have been four recognized HIV-1 cross species transmissions from primates to humans: groups M, N, O, and P. Group M is responsible for the global HIV-1 pandemic while groups N, O, and P viruses are extremely rare.

GPT-4 answer

HIV, the virus responsible for AIDS, has two main types: HIV-1 and HIV-2. Both types are transmitted through contact with the bodily fluids of someone who has HIV (blood, semen, vaginal fluids, anal fluids, and breast milk). However, they differ in their geographic spread, virulence, and progression to AIDS.

HIV-1 is the most widespread type globally and is more virulent and infectious than HIV-2. It is divided into four groups, each with a different geographic distribution and prevalence:

Group M (Major): This is the most common group worldwide, responsible for the majority of HIV infections. Group M is further subdivided into subtypes (or clades) labeled A through K, which have different prevalences in various parts of the world. For example, subtype B is the most common in North America and Europe, while subtype C is prevalent in southern Africa, India, and parts of Asia.

Group O (Outlier): This group is mainly found in West Central Africa and accounts for a small fraction of global HIV infections.

Group N (Non-M, Non-O): Identified in a limited number of individuals in Cameroon, Group N is extremely rare.

Group P: This group has been identified in only a few individuals, also primarily in Cameroon, and is very rare.

HIV-2 is less transmissible and usually progresses to AIDS more slowly than HIV-1. It is mainly found in West Africa but has been seen in other countries, particularly those with close ties to West Africa. HIV-2 is divided into eight groups, A through H:

Group A and B: These groups are the most common, with Group A being more prevalent and widespread. Group B has a more limited distribution.

Groups C to H: These groups are very rare, with each being identified in only a few cases, primarily in people from or linked to West Africa.

Prevalence

The prevalence of HIV-1 and HIV-2 varies significantly by region, with the highest rates of HIV-1 found in sub-Saharan Africa. HIV-2, while much less common globally, has its highest prevalence in West Africa. The global response to HIV has significantly impacted the prevalence of both types, with widespread access to HIV testing, treatment, and prevention methods helping to reduce the rate of new infections.

Exact numbers for the prevalence of each group and subgroup within HIV-1 and HIV-2 change as new data emerge and as efforts to combat HIV/AIDS evolve. For the most current statistics, it's advisable to consult the latest reports from the World Health Organization (WHO) and the Joint United Nations Programme on HIV/AIDS (UNAIDS).

Assessment

GPT-4 has knowledge about HIV-1, HIV-2 groups the prevalences around the world.

**Question 7 Explain all HIV-1 drugs and their drug class.**

Instruction sheet text with relevant information

There are about 30 FDA-approved ARVs. The most commonly used are: (1) the nucleoside RT inhibitors (NRTIs), including the cytosine analogs lamivudine (3TC) and emtricitabine (FTC), the tenofovir prodrugs tenofovir disoproxil fumarate (TDF) and tenofovir alafenamide (TAF), and zidovudine (AZT or ZDV) and abacavir (ABC); (2) the nonnucleoside RT inhibitors (NNRTIs), including nevirapine (NVP), efavirenz (EFV), rilpivirine (RPV), etravirine (ETR), and doravirine (DOR); (3) the pharmacologically boosted protease inhibitors (PIs), including ritonavir-boosted lopinavir (LPV; LPV/r) and ritonavir- or cobicistat boosted atazanavir (ATV; ATV/r or ATV/c) and darunavir (DRV; DRV/r or DRV/c); and (4) the first-generation integrase strand transfer inhibitors (INSTIs) raltegravir and elvitegravir, and the second-generation INSTIs dolutegravir (DTG), bictegravir (BIC), and cabotegravir (CAB). There are 4 FDA approved entry inhibitors. There is 1 FDA approved capsid (CA) inhibitor (CAI)– lenacapavir.

GPT-4 answer

HIV-1 (Human Immunodeficiency Virus Type 1) drugs are classified into several categories based on their mechanism of action against the virus. These drugs work at different stages of the HIV life cycle to prevent the virus from replicating and reduce the viral load in the body. Below is a comprehensive list of HIV-1 drug classes, along with examples of drugs within each class:

1. Nucleoside Reverse Transcriptase Inhibitors (NRTIs)

NRTIs block the reverse transcriptase enzyme, which the virus uses to make a DNA copy of its RNA. These drugs are often called "nucleoside analogs" because they are similar to the nucleosides used to make DNA but cause a termination of the DNA chain. Abacavir (ABC) Didanosine (ddI) Emtricitabine (FTC) Lamivudine (3TC) Stavudine (d4T) Tenofovir disoproxil fumarate (TDF) Zidovudine (AZT or ZDV)

2. Non-Nucleoside Reverse Transcriptase Inhibitors (NNRTIs)

NNRTIs bind to and inhibit the reverse transcriptase enzyme directly. This class of drugs does not compete with nucleosides, hence the name "non-nucleoside." Efavirenz (EFV) Etravirine (ETR) Nevirapine (NVP) Rilpivirine (RPV) Doravirine (DOR)

3. Protease Inhibitors (PIs)

PIs block the protease enzyme, which the virus needs to cut the newly made viral proteins into smaller pieces for assembling new HIV viruses. Atazanavir (ATV) Darunavir (DRV) Fosamprenavir (FPV) Lopinavir (LPV) Ritonavir (RTV) Saquinavir (SQV)

4. Integrase Strand Transfer Inhibitors (INSTIs)

INSTIs block the integrase enzyme, preventing the integration of the viral DNA into the host cell's DNA, a critical step in the viral replication cycle. Dolutegravir (DTG) Elvitegravir (EVG) Raltegravir (RAL) Cabotegravir (CAB)

5. Entry Inhibitors

This class of drugs prevents HIV from entering into the host cell by blocking the receptors on the cell's surface or by preventing the viral envelope from fusing with the cell membrane. CCR5 Antagonists Maraviroc (MVC) Fusion Inhibitors Enfuvirtide (T-20)

6. Post-Attachment Inhibitors

These drugs interfere with HIV's ability to interact with the CD4 receptors on the surface of T-cells after the virus has already attached to the cell. Ibalizumab (IBA)

7. Pharmacokinetic Enhancers

Pharmacokinetic enhancers are used in combination with other HIV drugs to increase their effectiveness by boosting their levels in the blood. Cobicistat (COBI) Ritonavir (RTV) (also a Protease Inhibitor but commonly used as a booster)

Combination Drugs

Many HIV medications are available as combination pills, which contain two or more drugs from the same or different classes. These combinations reduce the pill burden and simplify the treatment regimen, making it easier for patients to adhere to their therapy. Atripla (EFV/FTC/TDF) Truvada (FTC/TDF) Triumeq (ABC/DTG/3TC) Biktarvy (BIC/FTC/TAF)

This list covers the main classes and examples of drugs used to treat HIV-1. It's important to note that research and development in the field of HIV treatment are ongoing, and new drugs and drug classes may emerge.

Assessment

GPT-4 has knowledge about approved HIV-1 antiretrovirals, but missing the INSTI drug Bictegravir.

**Question 8 What kind of HIV treatment history information can be reported in a study that related to drug resistance test?**

Instruction sheet text with relevant information

Important information from a paper regarding treatment would include (1) How many patients received ART (ART-experienced) and how many had not (i.e., were ART-naïve); (2) How many ART-experienced patients received an ARV belonging to specific ARV classes; (3) Which drugs in each drug class were received among ART-experienced patients; and (4) How many ART regimens were received by a patient (i.e., the patient’s ART history).

GPT-4 answer

In studies related to drug resistance testing for HIV, several key pieces of treatment history information are typically reported. This information is crucial to understand the context of drug resistance and its implications for treatment options. Here are some of the types of treatment history information that may be included in such studies:

Baseline Antiretroviral Therapy (ART) Regimen: Details about the specific antiretroviral drugs the patient was receiving at the start of the study, including the class of drugs (e.g., NRTIs, NNRTIs, PIs, INSTIs) and the specific combination of medications.

Duration of ART: How long the patient has been on antiretroviral therapy, which can be critical for understanding the development of resistance. Longer durations of therapy might be associated with a higher likelihood of resistance mutations.

Adherence to Treatment: Information regarding how consistently the patient has been taking their ART medications, as poor adherence is a known risk factor for the development of drug resistance.

Previous ART Regimens: A history of all previous antiretroviral regimens the patient has been on, including any changes in medication due to side effects, treatment failure, or other reasons. This can help identify patterns of resistance.

Treatment Failures: Any instances where the patient's HIV infection was not adequately controlled by ART, including virological failure (where the virus is not suppressed to undetectable levels), immunological failure (decline in CD4 cell counts), or clinical failure (progression of HIV disease or the occurrence of HIV-related illnesses).

Resistance Mutations: Specific information about any known resistance mutations the virus has developed, either currently or in the past, which can influence the choice of future treatment options.

Viral Load and CD4 Counts: The levels of HIV RNA in the blood (viral load) and the counts of CD4 cells at various points throughout treatment, as these are key indicators of treatment effectiveness and the health of the immune system.

Co-Infections: Information on any co-infections the patient may have, such as hepatitis B or C, since these can impact HIV treatment choices and outcomes.

Pharmacogenetic Information: Details about any genetic factors that might affect the patient's metabolism of HIV drugs, which can influence drug effectiveness and the risk of side effects.

This information helps researchers and clinicians understand the effectiveness of current treatments, identify factors contributing to drug resistance, and guide the development of new strategies to overcome resistance. It's also crucial for tailoring treatment plans to individual patients to maximize the effectiveness of ART and improve health outcomes.

Assessment

GPT-4 has knowledge about the treatment history and the factors that related to drug resistance.

**Question 9 What methods were used for HIV phenotypic testing?**

Instruction sheet text with relevant information

Phenotypic testing is usually performed on site-directed mutants, virus isolates that developed mutations during in vitro passage experiments, or on clinical viruses samples obtained directly from a patient sample. If phenotypic testing was performed, there are usually one or more sentences describing the phenotypic test method. The most common method is the PhenoSense assay (which can also be referred to as PhenoSenseGT).

GPT-4 answer

HIV phenotypic testing, particularly for antiretroviral drug resistance, involves directly measuring the ability of HIV to grow in the presence of antiretroviral drugs. This method assesses the susceptibility of the virus to various antiretroviral agents, which is critical for guiding therapy in individuals with HIV, especially those with drug-resistant HIV strains. Here are some of the methods used:

Viral Culture: This method involves culturing the patient's virus in the presence of different concentrations of antiretroviral drugs. By measuring the concentration of drug required to inhibit viral replication by 50% (IC50), the sensitivity of the virus to each drug can be determined. This method can be labor-intensive and time-consuming.

Recombinant Viral Phenotyping: This is a more modern approach and involves creating recombinant viruses that contain the patient's HIV envelope or reverse transcriptase and protease genes. These recombinant viruses are then tested against various antiretroviral drugs to determine their susceptibility. This method is faster and more standardized than traditional viral culture techniques. It includes specific assays such as the PhenoSense™ assay.

Genotypic-Phenotypic Correlation Models: Although not a direct phenotypic testing method, these computational models predict phenotypic resistance based on genotypic data (the genetic mutations present in the viral genome). These models are based on large databases of genotypic and phenotypic data, allowing for predictions of drug resistance without the need for culture-based phenotypic assays.

Phenotypic resistance testing provides precise information about the resistance of HIV to specific antiretroviral drugs, but due to its higher cost and longer turnaround times compared to genotypic testing, it is usually reserved for complex cases where genotypic testing is insufficient for guiding therapy decisions.

Assessment

GPT-4 has knowledge about how to conduct the HIV-1 phenotypic testing.

**Question 10 How is it possible to know whether a paper is reporting HIV patients from clinical trials?**

Instruction sheet text with relevant information

It is often useful to know if the patients in a study were part of a clinical trial because in such studies the ART histories of the patients are more likely to be less heterogeneous.

GPT-4 answer

To determine whether a paper is reporting HIV patients from clinical trials, there are specific aspects you can look for:

Title and Abstract: The title may often include terms like "clinical trial," "study," "randomized," or "intervention." The abstract should summarize the nature of the study, including whether it was a clinical trial involving HIV patients.

Methods Section: This is the most detailed section where the type of study is described. Look for information on how patients were enrolled, the kind of intervention they received, and how the data was collected and analyzed. Terms like "randomization," "placebo-controlled," "double-blind," or "phase I/II/III" indicate a clinical trial.

Participant Information: This section should provide details about the HIV patients, including inclusion and exclusion criteria, demographic information, and the number of participants.

Ethics Statement: Clinical trials will have an ethics statement mentioning the approval from an Institutional Review Board (IRB) or Ethics Committee and details about patient consent.

Trial Registration: Many journals require that clinical trials be registered in a public trials registry, such as ClinicalTrials.gov. The registration number should be listed in the paper.

Results: This section should report on the outcomes measured, statistical analyses performed, and whether the study met its endpoints.

Discussion and Conclusion: The authors will discuss the implications of the trial outcomes and how it adds to the current understanding of HIV treatment or management.

References: Clinical trial papers will often cite other clinical trials, protocols, or methodological papers.

If the paper you are referring to is digital, you can search for these sections using keywords related to clinical trials. If it is not digital, as in the case of the image you've uploaded, you would manually check for these indicators within the paper. If you'd like, I can take a look at the image to help identify if it is indeed reporting on HIV patients from clinical trials.

Assessment

GPT-4 has knowledge about determining if the subjects in the paper were in clinical trials.
